# Lineage tracking to reveal the fate of hematopoietic stem cells influenced by Flk2^−^ multipotent progenitors after transplantation

**DOI:** 10.1038/s12276-022-00922-w

**Published:** 2023-01-13

**Authors:** Zheng Wang, Du Jiang, Mary Vergel-Rodriguez, Anna Nogalska, Rong Lu

**Affiliations:** 1 Department of Stem Cell Biology and Regenerative Medicine, Eli and Edythe Broad Center for Regenerative Medicine and Stem Cell Research, Keck School of Medicine, University of Southern California, Los Angeles, CA 90033 USA; 2 Present Address: Jinfeng Laboratory, Chongqing, 401329 China; 3 Medical Center of Hematology, Xinqiao Hospital, Army Medical University, Chongqing, 400037 China

**Keywords:** Haematopoietic stem cells, Differentiation

## Abstract

After transplantation, hematopoietic stem cells (HSCs) sustain blood cell regeneration throughout the patient’s life. Recent studies suggest that several types of mature blood cells provide feedback signals to regulate HSC fate. However, the potential feedback effect of hematopoietic progenitor cells has not been characterized to date. The present investigation demonstrated that multipotent progenitors (MPPs) promoted T cell production of HSCs when both cell types were cotransplanted in mice. Using genetic barcodes to track individual HSCs in mice, we found that the increased T cell production by HSCs was associated with the combined effects of altered lineage bias and clonal expansion during HSC differentiation. We showed that MPP and HSC co-transplantation promoted the multilineage differentiation of HSCs in the short term while preserving lymphoid-specialized HSC differentiation in the long term. Our findings indicate that MPPs derived from HSCs regulate the fate of HSCs after bone marrow transplantation.

## Bone marrow transplants: Tracking the fate of stem cells

Genetic barcoding technology reveals how a specific group of progenitor cells regulates the fate of hematopoietic stem cells (HSCs), the precursors of blood cells, following bone marrow transplantation. HSCs are used in bone marrow transplantation to boost blood regeneration in leukemia patients. HSCs differentiate into different groups of mature blood cells, which then form a ‘feedback loop’, influencing the fate of HSCs. The first descendants of HSCs are multipotent progenitor cells (MPPs), which are frequently transplanted alongside HSCs. Exactly how MPPs influence HSC differentiation is unclear. Rong Lu at the University of Southern California, Los Angeles, USA, and co-workers used genetic barcoding technology to track individual HSCs when they were transplanted alongside MPPs into mice. One specific group of MPPs boosted long-term T-cell production from HSCs, and also regulated the fate of HSCs over time.

## Introduction

Hematopoietic stem cells (HSCs) reside in specialized bone marrow microenvironments and replenish blood and immune system cells in the body. HSCs can undergo several fates, including apoptosis, quiescence, migration, differentiation, and self-renewal. Previous studies have shown that these HSC fates are largely regulated by the hematopoietic microenvironment, including niche cells in this microenvironment^1,2^. During hematopoiesis, HSCs replenish multipotent progenitors (MPPs), which subsequently differentiate into specialized myeloid and lymphoid progenitors, producing all types of mature blood cells through stepwise differentiation^3,4^. There has been extensive research on the regulation of HSC fates by nonhematopoietic niche cells, such as mesenchymal stromal cells surrounding vessels^2,5^, endothelial cells^6^, adipocytes^7^, and osteoblasts^8^. Although hematopoietic progenitor cells (HPCs) are localized in bone marrow, little is known about their effects on HSC fate.

Recent evidence has demonstrated that several mature progenies of HSCs are constituents of the HSC niche, including megakaryocytes^9,10^ and regulatory T cells^11^, which regulate HSC behavior. Notably, alternatively polarized M2 macrophages and classical M1 macrophages promoted and inhibited HSC self-renewal through the differential expression of Arg1 and NOS2 ex vivo, respectively^12^. Furthermore, depletion of common lymphoid progenitors (CLPs) caused by the ablation of osteoblasts has been shown to alter HSC maintenance^13^. In the hematopoietic hierarchy, fetal liver kinase 2 (Flk2)^-^ MPPs are the first downstream progenitors of HSCs and may directly interact with HSCs and the HSC niche. However, whether these MPPs influence the fate of HSCs remains unknown.

For clinical HSC transplantation, a mixture of long-term HSCs and short-term HPCs, including MPPs^14,15^, are infused in patients with various blood diseases, such as leukemia^16^ and multiple myeloma^17^, to regenerate their blood and immune systems. It is also performed during organ transplantation to eliminate immune responses^18^. Once transplanted, HSCs and HPCs undergo heterogeneous differentiation to produce various types of blood cells^19–25^. Individual HSCs show distinct differentiation biases toward myeloid or lymphoid lineages^26^. Some HSCs produce balanced multilineage progenies^4,23,27^. Notably, lineage-biased HSCs have been found after either inflammatory stimulation or native hematopoiesis^28^. In addition to their heterogeneous capacity for self-renewal and differentiation, individual HSCs exhibit changes in differentiation programs at various transplantation doses^29^. For example, high-dose HSC transplantation resulted in short-term HSC differentiation and exhaustion^29^.

A better understanding of the fate regulation of transplanted HSCs will provide new insight into HSC-based clinical therapy. Currently, population-level lineage tracking methods are widely used, but these approaches do not provide cellular heterogeneity information. In this study, we tracked the clonal differentiation of individual HSCs using cell barcoding technology. Specifically, we combined lentiviral delivery of highly diverse genetic barcodes with high-throughput sequencing. Compared to the other tracking methods using viral insertion site or transposon tagging^30,31^, this technology exhibits high sensitivity and allows precise quantification. Additionally, this technology shows the high-throughput capacity to simultaneously track many HSCs in a single mouse. In this study, using the genetic barcode tracking technique, we showed that Flk2^−^ MPPs regulate HSC fate at the clonal level.

## Materials and methods

### Mice

All donor and recipient mice were 8–12 weeks old at the time of transplantation. C57BL6/J mice (CD45.2^+^, Jackson Laboratory, stock #00664) were the recipient mice. Donor HSCs were derived from C57BL6/J mice (CD45.1^+^, Jackson Laboratory, stock #002014). F1 mice (CD45.1^+^/CD45.2^+^) derived from the cross of the two aforementioned mouse strains were used to isolate donor MPPs that were cotransplanted with HSCs. In a replicated experiment (Supplementary Fig. 4), F1 (CD45.1^+^/CD45.2^+^) mice were donors for HSCs, and C57BL6/J (CD45.1^+^) mice were donors for MPPs. C57BL6/J (CD45.2^+^) mice were recipients. Helper cells were extracted from the same strain as the recipient mice. Therefore, all the helper cells carried the same CD45 allele markers as the recipients. All mice were subjected to 950 cGy irradiation before transplantation. Mice were bred and housed at the Research Animal Facility of the University of Southern California or Stanford University. Animal procedures were approved by the respective Institutional Animal Care and Use Committees at the University of Southern California and Stanford University.

### Cell isolation and transplantation

A total of 3000 HSCs (lineage (CD3, CD4, CD8, B220, Gr1, Mac1, Ter119) ^−^/ckit^+^/Sca1^+^/Flk2^−^/CD34^−^/CD150^+^) and 30,000 MPPs (lineage (CD3, CD4, CD8, B220, Gr1, Mac1, Ter119) ^−^/IL7Ra^−^/ckit^+^/Sca1^+^/Flk2^−^/CD34^+^) were extracted from crushed bones of donor mice, enriched with CD117 microbeads (AutoMACS, Miltenyi Biotec, Auburn, CA), and isolated using double FACS sorting with a FACS-Aria II flow cytometer (BD Biosciences, San Jose, CA). HSCs were transduced by lentivirus carrying barcodes and GFP for 13–15 h and then transplanted into recipient mice with or without MPPs via retro-orbital injection. HSC clonal labeling was performed as described previously^32,33^. In addition to donor HSCs and MPPs, 250,000 unfractionated bone marrow cells (helper cells) flushed from the femur were transplanted into each mouse.

### Collection of blood samples and FACS

Blood collected from a small transverse cut in the tail vein was added to phosphate-buffered saline (PBS) supplemented with 10 mM EDTA and treated with 2% dextran at room temperature for 30 min to remove the red blood cells. The collected samples were lysed on ice using ACK lysis buffer (150 mM NH4Cl, 1 mM KHCO3, and 0.1 mM EDTA) for 5 min to further remove the residue red blood cells. The samples were then incubated with mixed antibodies as described previously^29,32,34^ for 30 min at 4 °C and finally resuspended in PBS supplemented with 2% FBS and DAPI. Antibodies were purchased from eBioscience (currently Life Technologies/Thermo Fisher) and BioLegend, as indicated in our previous reports. The cells were sorted using BD FACS-Aria I and II flow cytometers into granulocyte, B, CD4 T, and CD8 T cell fractions. The CD45 marker was analyzed to distinguish donor and recipient blood cells. Data obtained via flow cytometry were analyzed using Diva software 8.0.1 (BD Biosciences, San Jose, CA). The following cell surface markers were used to harvest 4 types of hematopoietic cell populations:

Granulocytes: CD4^−^/CD8^−^/B220^−^/CD19^−^/Mac1^+^/Gr1^+^/side scatter ^high^;

B cells: CD4^−^/CD8^−^/Gr1^−^/Mac1^−^/B220^+^/CD19^+^;

CD4 T cells: B220^−^/CD19^−^/Mac1^−^/Gr1^−^/TCRab^+^/CD4^+^/CD8^−^; and

CD8 T cells: B220^−^/CD19^−^/Mac1^−^/Gr1^−^/TCRab^+^/CD4^−^/CD8^+^.

### DNA barcode extraction and quantification

Genomic DNA was extracted from sorted hematopoietic cells and amplified using Phusion PCR Master Mix (Thermo Scientific, Waltham, MA). The polymerase chain reaction (PCR) was stopped at the exponential phase, and the PCR product was purified using AMPure XP beads (Beckman Coulter A63880) before high-throughput sequencing. The sequencing data were analyzed as previously described^29,33,34^. Barcode data were analyzed using our customized Python scripts^32–34^. We filtered out clones with more than 0.5% white blood cell (WBC) abundance in one cell type at one-time point and absent in all other cell types and time points. Sequencing data were combined with FACS data to calculate the clonal abundance as follows:

Clonal abundance = 100% × (each cell population (Gr, B, CD4 T, or CD8 T cells)% WBCs) × (donor% each cell population) × (GFP% donor cells) × (number of reads for each barcode)/(total reads of all barcodes).

### DNA barcode analysis

Clone clustering was performed based on the clonal abundance of all cell types and time points. A principal component analysis was performed, and 12 principal components were summarized using UMAP dimensionality reduction^35^ after combining the data for each clone from all the samples. Next, the ‘FindNeighbors’ and ‘FindClusters’ functions in Seurat^36^ were used to cluster the barcode data. UMAP plots were generated using the “DimPlot” function in Seurat, and 10 distinct clusters were obtained. The relative abundance of the barcodes between myeloid cells and lymphoid cells was analyzed to classify lineage-biased and specialized HSC clones. A lineage-biased clone was defined as a clone in which the relative copy number in one lineage was greater than 2.4142 (cotangent 22.5°) fold of the relative copy number in the other lineage. A lineage-specialized clone was defined as a clone that produced a measurable amount of lymphoid or myeloid cells but not cells of both lineages (<0.01% WBCs)^29,34^.

### Statistical analysis

The data are shown as the mean ± SEM. The statistical analysis was carried out using one-tailed and two-sample unequal variance Student’s *t* tests for two groups with Microsoft Excel 2016. Statistical significance is reported as *P ≤ 0.05 and **P < 0.01. Violin plots and heatmaps were generated using the ggplot2 (https://cran.r-project.org/web/packages/ggplot2/) and pheatmap (https://cran.r-project.org/web/packages/pheatmap/) packages, respectively. The Shannon index was calculated for each cell type and time point per mouse using the Vegan package according to the user manual (https://github.com/vegandevs/vegan).

## Results

### Cotransplantation with MPPs promotes HSCs to produce more T cells

**Fig. 1:**
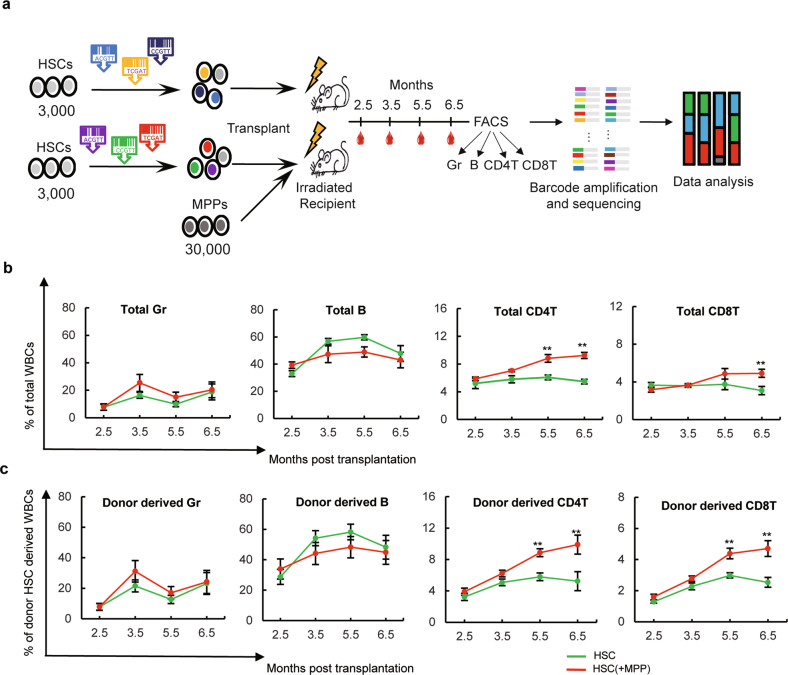
The dynamics of blood cell production after MPP cotransplantation. **a** Experimental workflow. A total of 3000 barcoded HSCs with or without 30,000 MPPs were transplanted into each irradiated recipient mouse. Peripheral blood was collected from recipient mice at four-time points after transplantation and sorted into granulocytes (Gr), B cells, CD4 T cells, and CD8 T cells for population and clonal level analyses. **b** Abundance of granulocytes (Gr), B cells, CD4 T cells, and CD8 T cells in the peripheral blood of the recipient mice. **c** Abundance of granulocytes (Gr), B cells, CD4 T cells and CD8 T cells in the donor-derived white blood cell (WBC) sample. *n* = 5 mice for the HSC-only (HSC) group and *n* = 7 mice for the HSC cotransplanted with MPP (HSC(+MPP)) group in the population level analysis. The data are shown as the mean ± SEM. **P < 0.01 for the comparison of the two experimental groups as determined by one-tailed Student’s *t* test. WBCs white blood cells.

To investigate whether MPPs influence the differentiation of HSCs, we used genetic barcoding technology^23,33^ to track the blood production of individual HSCs (Fig. 1a). We purified mouse HSCs (lineage (CD3, CD4, CD8, B220, Gr1, Mac1, Ter119) ^−^/ckit^+^/Sca1^+^/Flk2^−^/CD34^−^/CD150^+^) and labeled them with genetic barcodes as previously described^29,32,33^. We then transplanted 3000 HSCs with or without 30,000 MPPs (lineage (CD3, CD4, CD8, B220, Gr1, Mac1, Ter119) ^−^/IL7Ra^−^/ckit^+^/Sca1^+^/Flk2^−^/CD34^+^) into a lethally irradiated mouse (Supplementary Fig. 1). The four most abundant white blood cells in the peripheral blood, namely, granulocytes (Gr), B cells, CD4 T cells, and CD8 T cells, were sorted at different time points after transplantation to investigate HSC differentiation (Fig. 1a, Supplementary Fig. 2). Flow cytometry analysis revealed that the addition of MPPs exerted no effect on Gr or B cell production at any timepoint (Fig. 1b, c). However, it significantly increased the production of all T cells and donor HSC-derived T cells 5.5 and 6.5 months post-transplantation when HSCs were cotransplanted with MPPs (Fig. 1b, c). In addition, MPP cotransplantation did not exert a significant effect on HSC-derived donor chimerism in any measured blood cell type (Supplementary Fig. 3). We switched the donor and recipient mouse strains and obtained similar results (Supplementary Fig. 4). These data suggest that cotransplantation of MPPs results in greater long-term T cell production after transplantation (Fig. 1).

### Changes in the blood cell differentiation of HSC clones after cotransplantation with MPPs

**Fig. 2:**
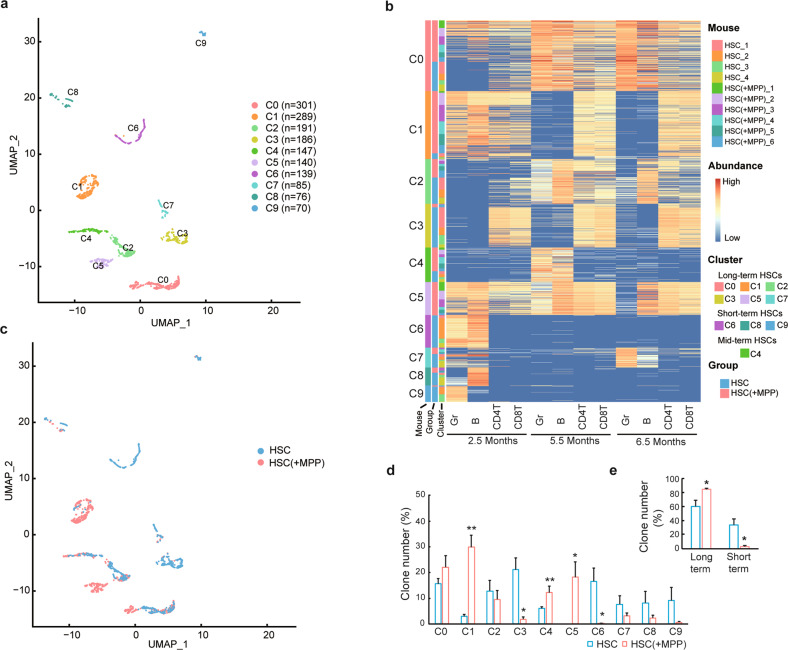
HSC differentiation after MPP cotransplantation. **a** UMAP plot showing unsupervised HSC clustering. Colors highlight distinct HSC clusters. **b** Heatmap depicting the number of granulocytes (Gr), B cells, CD4 T cells, and CD8 T cells produced by individual HSC clones. Color shows clonal abundance (% white blood cells) after log_2_ normalization. **c** UMAP plot showing distinct HSC clusters that consist of HSCs from different experimental groups. **d** Number of clones derived from different experimental groups in each cluster. **e** Number of clones derived from different experimental groups in long-term clusters (C0–3, 5, and 7) and short-term clusters (C6, 8, and 9). The data are shown as the mean ± SEM. *n* = 4 mice for the HSC-only (HSC) group and *n* = 6 mice for the HSC cotransplanted with MPP (HSC(+MPP)) group in the clonal level analyses of all figures. *P ≤ 0.05 and **P < 0.01 for the comparison of the two experimental groups as determined by one-tailed Student’s *t* test.

To systematically determine how MPPs influence the differentiation of HSC clones, we performed unbiased clustering of all barcoded HSC clones based on the abundances of the four blood-cell types at three-time points. We identified ten separate clusters via the uniform manifold approximation and projection (UMAP) algorithm (Fig. 2a, b). According to the timing of blood reconstitution, these ten HSC clusters were classified into long-term (C0–C3, C5, and C7), middle-term (C4), and short-term (C6, C8, and C9) categories. Long-term clusters represented sustained hematopoietic reconstitution for up to 6.5 months post-transplantation, while short-term clusters represented HSCs that produced blood cells only at 2.5 months post-transplantation. Middle-term clusters represented HSCs that produced blood cells at 5.5 months but not at 2.5 or 6.5 months post-transplantation.

We found that the fraction of HSC clones from the MPP cotransplantation group and the HSC-only group significantly differed across multiple clusters. In general, the abundance of the long-term and middle-term HSCs significantly increased (C4), whereas the abundance of short-term HSCs significantly decreased with MPP cotransplantation (Fig. 2c–e). For example, cotransplantation with MPPs significantly increased the number of HSC clones in Clusters C1, C4, and C5 and significantly reduced the number of HSC clones in Clusters C3 and C6 (Fig. 2d). The significant reduction in short-term HSC Cluster C6, together with the reduction in other short-term clusters, such as C8 and C9, as well as an increase in the corresponding middle-term Cluster C4, suggested that MPP cotransplantation prevented the HSC clones from committing to short-term blood cell production (Fig. 2b, d).

After transplantation, HSCs in the C1 and C3 clusters showed biased differentiation toward T cells in the long term, and they differed in their short-term blood cell production levels (Fig. 2b). The difference between the C1 and C3 clusters suggested that during short-term blood cell production, MPP cotransplantation promoted HSC clones into multilineage blood differentiation (C1) and prevented exclusive T cell production (C3). In addition, the fraction of HSC clones from the MPP cotransplantation group was significantly increased in the long-term multilineage differentiation cluster (C5) (Fig. 2d). Taken together, these data suggest that MPP cotransplantation inhibited the short-term blood cell production and promoted multilineage blood cell differentiation of HSCs (Fig. 2).

### HSC clones’ contribution to T cells changes with MPP cotransplantation

**Fig. 3:**
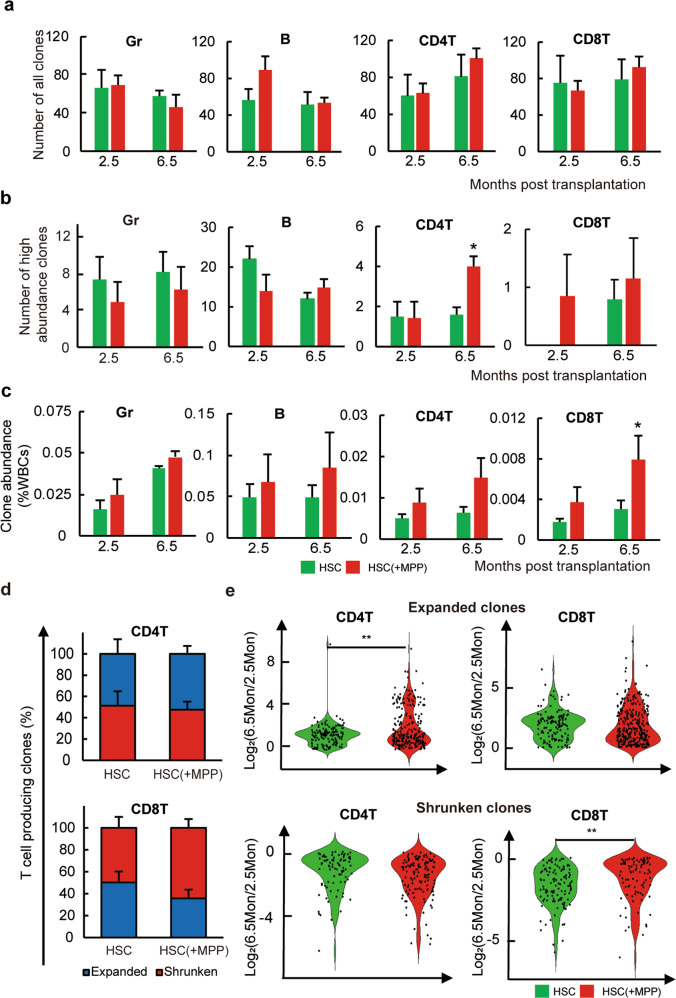
MPP cotransplantation influences T cell production of HSC clones. **a** Number of clones producing granulocyte (Gr), B, CD4 T, and CD8 T cells at 2.5 and 6.5 months post transplantation. **b** Number of high abundance clones (above 0.05% of WBCs) producing each cell type. **c** Average abundance of clones producing each cell type. **d** Proportion of “shrunken” and “expanded” clones among all persisted clones that produce CD4 T and CD8 T cells. The ratio of CD4 T or CD8 T cell production at 6.5 months versus 2.5 months post transplantation defined clones as “shrunken” (below 1) and “expanded” (above 1). **e** Clonal abundance changes over time for “expanded” or “shrunken” clones that produce CD4 T or CD8 T cells. Each dot represents one clone. Data are shown as the mean ± SEM. *P ≤ 0.05 and **P < 0.01 for the comparison of the two experimental groups as determined by one-tailed Student’s *t* test. WBCs white blood cells.

To better understand how MPP cotransplantation influences the T cell differentiation of HSC clones in the long term, we investigated changes in the blood cell production of HSC clones between 2.5 and 6.5 months post-transplantation since HSC blood production was similar at 5.5 and 6.5 months post-transplantation (Fig. 2b, Supplementary Fig. 8). We found that the total number of HSC clones contributing to each blood cell type and the clonal distribution of the blood cell contribution did not significantly change with MPP cotransplantation (Fig. 3a, Supplementary Fig. 5, Supplementary Tables 1–2). However, the number of highly abundant clones producing CD4 T cells significantly increased 6.5 months post-transplantation (Fig. 3b). Moreover, the average contribution of HSC clones to the CD8 T cell population significantly increased 6.5 months post-transplantation (Fig. 3c). These data suggest that an increase in T cells associated with MPP cotransplantation was a result of increased T cell production by HSC clones but not related to the number of HSC clones that differentiated into T cells.

Comparing the changes in HSC clones’ blood cell production between 2.5 and 6.5 months post-transplantation, we identified ‘expanded’ and ‘shrunken’ clones that increased or decreased their blood cell production over time, respectively. The number of these expanded and shrunken clones of all the blood cell types that we examined were not significantly different after the addition of MPPs (Fig. 3d, Supplementary Fig. 6). However, the contribution of these clones to the T cell population significantly differed (Fig. 3e). With MPP cotransplantation, the CD4 T cell production of the expanded HSC clones was significantly increased, whereas CD8 T cell production of the shrunken HSC clones was significantly decreased over time (Fig. 3e). These data suggest that MPP cotransplantation influenced specific subsets of HSCs in their differentiation into T cells (Fig. 3).

### MPP cotransplantation alters the lineage bias of HSC clones

**Fig. 4:**
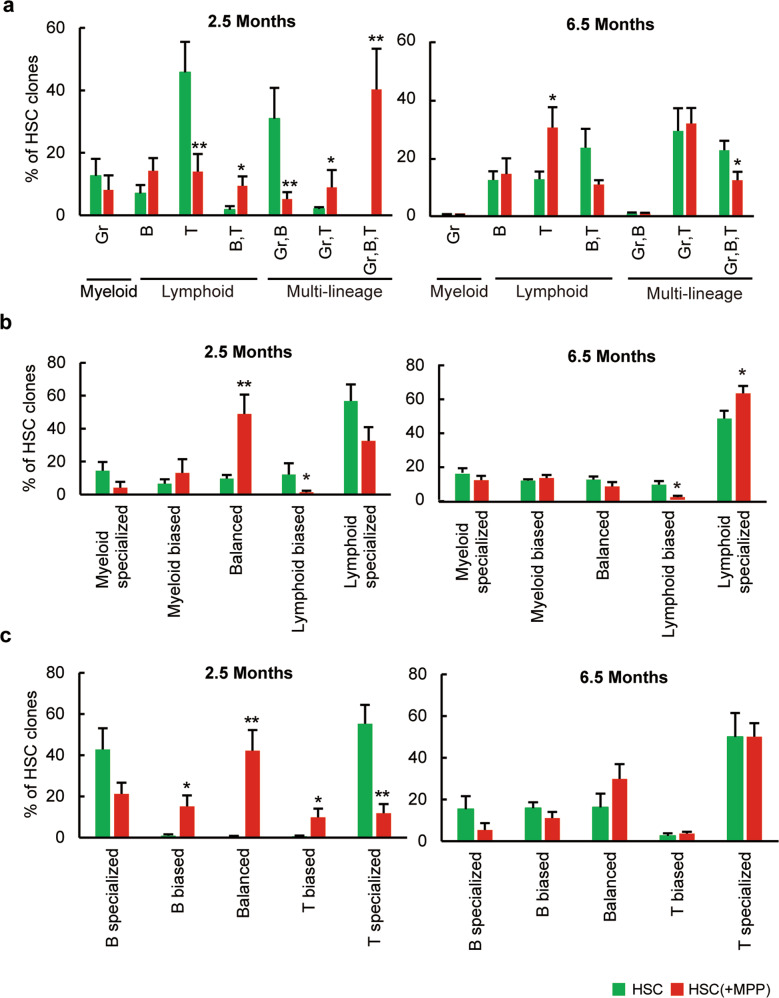
MPP cotransplantation alters HSC lineage bias. **a** Number of HSC clones producing different cell types. Clones are categorized by the combination of cell types that they produce 2.5 or 6.5 months post-transplantation. All combinations of the quantified cell types are displayed in the graph. Clones that produced CD4 T or CD8 T cells were combined as T cell-producing clones. **b** Number of HSC clones exhibiting various lineage biases. A lineage-biased clone is defined as a clone in which the relative copy number in one lineage is greater than 2.4142 (cotangent of 22.5°) fold of the copy number in the other lineage. A lineage-specialized clone produces measurable numbers of lymphoid or myeloid cells but not cells of both lineages. **c** Number of HSC clones exhibiting various degrees of B cell and T cell biases. All clones producing B cells and T cells are included in this analysis. The definition of biased, balanced, and specialized clones is similar to that described in (**b**). The data are shown as the mean ± SEM. *P ≤ 0.05 and **P < 0.01 for the comparison of the two experimental groups as determined by one-tailed Student’s *t* test.

To determine how MPPs influence the lineage preference of HSC differentiation, we analyzed the contribution of individual HSC clones to the four blood cell types. At 2.5 months post-transplantation, MPP cotransplantation resulted in a significantly higher number of multilineage clones and significantly fewer T cell lineage specialized clones (Fig. 4a, Supplementary Fig. 7). In contrast, at 6.5 months post-transplantation, MPP cotransplantation resulted in a significantly lower number of multilineage clones and a significantly higher number of specialized T cell clones (Fig. 4a, Supplementary Fig. 7). These results suggest that the addition of MPPs changed the lineage preference of HSC differentiation in the short term and long term.

We classified HSC clones based on their lineage bias as previously described^29,32,34^. We found that MPP cotransplantation resulted in a significantly higher number of lineage-balanced clones 2.5 months post-transplantation (Fig. 4b). At 6.5 months post-transplantation, MPP cotransplantation resulted in significantly more lymphoid-specialized clones (Fig. 4b). Lymphoid-specialized clones exclusively contribute to lymphoid lineages, while lymphoid-biased clones contribute to multiple lineages but show a tendency to contribute more to lymphoid lineages. Altogether, these data suggest that MPP cotransplantation promoted multilineage and balanced differentiation of HSCs in the short term and T-lymphoid-specialized production in the long term (Fig. 4a, b).

Furthermore, we analyzed the contribution of HSCs to B cell and T cell lineages. B and T cell balanced clones were defined as clones producing similar fractions of B cells and T cells^29,32,34^. At 2.5 months post-transplantation without MPPs, all B cell and T cell-producing clones showed lineage specialization by producing B cells or T cells but not both cell types (Fig. 4c). In contrast, MPP cotransplantation resulted in a significantly higher number of B cell biased clones, balanced clones, and T cell biased clones and in a significantly lower number of T cell-specialized clones 2.5 months post-transplantation (Fig. 4c). At 6.5 months post-transplantation, no significant difference was found between B cell and T cell biases associated with MPP cotransplantation (Fig. 4c). These data suggest that MPP cotransplantation promoted the production of multiple lymphoid cell types in the short term.

### MPP cotransplantation promotes persistent HSC blood cell differentiation

**Fig. 5:**
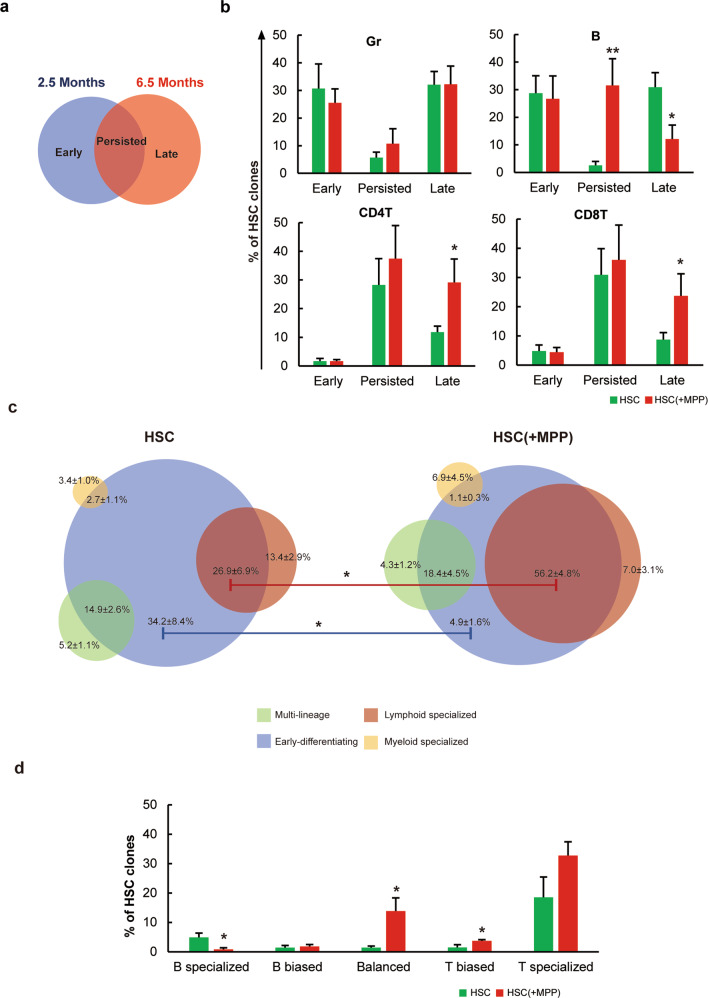
MPP cotransplantation promotes persistent HSC blood cell production. **a** Diagram illustrating the definition of early, late, and persisted clones based on their blood cell production 2.5 and 6.5 months post-transplantation. **b** Percentage of all detected barcoded HSC clones that produced granulocytes (Gr), B, CD4 T cells, and CD8 T cells at either 2.5 or 6.5 months post-transplantation. **c** Venn diagram showing the fraction of early differentiating clones that exhibited various degrees of lineage bias 6.5 months post-transplantation. **d** Bias between B and T cell production for clones that persisted and exhibited lymphoid specialization as defined in (**c**). *P ≤ 0.05 and **P < 0.01 for comparison of the two experimental groups as determined by one-tailed Student’s *t* test.

We analyzed changes in the blood cell production of HSC clones over time by comparing data obtained at 2.5 and 6.5 months post-transplantation, as HSC blood cell production was similar 5.5 and 6.5 months post-transplantation (Fig. 1, Fig. 4, Supplementary Fig. 8). We classified HSC clones into ‘persisted’, ‘early’ and ‘late’ clones. The persisted clones produced blood cells at both 2.5 and 6.5 months post-transplantation, while early and late clones produced blood cells at 2.5 or 6.5 months post-transplantation, respectively (Fig. 5a). We did not find any significant change in the number of myeloid-producing clones over time with MPP cotransplantation; however, the number of lymphoid-producing clones changed significantly over time (Fig. 5b). Specifically, the number of HSC clones that persisted in producing B cells significantly increased with MPP cotransplantation, whereas the number of late clones producing B cells significantly decreased. Moreover, the number of late clones producing CD4 T or CD8 T cells significantly increased with MPP cotransplantation (Fig. 5b). These data suggest that the addition of MPPs promoted the persistent production of B cells and the long-term production of T cells by HSCs (Fig. 5a, b).

To determine whether and how early differentiating HSC clones produce different types of blood cells in the long term, we analyzed the overlap of the month 2.5 differentiating clones with the clones exhibiting multilineage, myeloid-specialized, and lymphoid-specialized differentiation at 6.5 months post-transplantation (Fig. 5c). We found that with MPP cotransplantation, early differentiating clones were significantly more likely to produce blood cells at a later time point (Fig. 5c). In particular, clones with MPP cotransplantation were significantly more likely to become lymphoid-specialized clones (Fig. 5c). Further analysis of these persisted lymphoid-specialized clones revealed that they produced a significantly higher number of B cell and T cell balanced and T-biased clones and a significantly lower number of B-specialized clones (Fig. 5d). These data suggest that MPP cotransplantation promoted early differentiating HSC clones to persistently produce T cells in the long term.

## Discussion

Previous studies have suggested that progeny cells terminally differentiated from stem cells in the intestine^37^ or skin^38^ form specialized niches that regulate stem cell quiescence. Nonhematopoietic cells, including mesenchymal stromal cells surrounding vessels^2,5^, endothelial cells^6^, adipocytes^7^, and osteoblasts^8^, have been proposed to be components of the HSC niche in the bone marrow microenvironment. Studies have also demonstrated that megakaryocytes residing in bone marrow microenvironments, progeny cells derived from HSCs, are HSC niche cells^9,10^. In this study, we showed that MPPs derived from stem cells may induce feedback regulation of stem cell differentiation. However, MPPs may not regulate HSCs through direct cell‒cell interactions and instead may interact with HSC niches. Future studies on the spatial interactions of MPPs with HSCs and HSC niches may provide new insights into the regulatory mechanism.

In addition to niche interactions, immune rejection is another possible mechanism of HSC differentiation regulation. Our data showed that cotransplanted MPPs were mostly exhausted within 3.5 months of transplantation (Supplementary Fig. 3a), which was consistent with previous studies in the field. Moreover, no significant increase in T cell production was observed 2.5 or 3.5 months post-transplantation (Fig. 1b, c). Therefore, the increase in lymphoid specialized cells 6.5 months post-transplantation (Fig. 4) was not likely to have been caused by immune rejection.

Due to the functional heterogeneity of HSCs, conventional technologies used to analyze cell populations are not suitable to study the MPP influence on distinct subsets of HSC clones. To reveal the molecular mechanisms underlying the distinct responses of HSC clones to MPP cotransplantation as shown in our study (Figs. 2–5), future studies integrating cellular barcoding technology and massively parallel single-cell RNA sequencing could enable simultaneous investigations into clonal trajectories and corresponding molecular profiles^39^.

Our previous study revealed that HSCs exhibited differentiation program adaptation in response to transplantation dose^29^. For instance, high HSC transplantation doses produced fewer T cell-specialized HSC clones^29^. In the present study, the number of T cell-specialized HSC clones was also significantly decreased at the early point, namely, 2.5 months post-transplantation with MPPs (Fig. 4a, b). This finding indicates that the addition of MPPs led to short-term effects that were similar to the addition of HSCs. Similar to high-dose HSC transplantation^29^, HSC and MPP cotransplantation produced significantly more lymphoid-balanced HSC clones, which persisted in producing balanced lymphoid cell production in the long term (Fig. 5d).

Flk2, also known as FLT3, is a receptor tyrosine kinase expressed on the surface of HSPCs. Flk2 is crucial for stem cell function and immune system development^40^. High levels of Flk2 expression have been associated with a high risk of relapse in pediatric AML patients^41^. The overexpression of Flk2 in AML blasts has been associated with a worsened prognosis^42^. Furthermore, Flk2 deficiency in mice significantly reduced multipotent, lymphoid, and myeloid progenitors, such as MPPs, CLPs, Pro-B, and B cells but not T cells^43^. In the hematopoietic hierarchy, Flk2 (fetal liver kinase 2)^-^ MPPs are the first downstream progenitors of HSCs^44^.

HSC transplantation is the only curative treatment for leukemia, and morbidity and mortality are associated with inefficient replenishment of blood cell lineages^45,46^. Our study showed that MPP cotransplantation promoted HSC-producing T cells via increased T-biased clone expansion and T-biased differentiation. An increase in myeloid bias and a reduction in lymphoid bias are hallmarks of HSC aging^47^. Therefore, the present research may have potential implications for treating hematopoietic aging^48^.

## Supplementary information

Supplemental Materials
