# Supplementary material for: Lineage tracking to reveal the fate of hematopoietic stem cells influenced by Flk2− multipotent progenitors after transplantation
Source: Exp Mol Med. 2023 Jan 13;55(1):205–14. doi: 10.1038/s12276-022-00922-w (PMC9898540; doi:10.1038/s12276-022-00922-w)
Supplement: Supplementary file 1 — Supplemental Materials [file 12276_2022_922_MOESM1_ESM.pdf]

Supplementary Materials

Lineage tracking to reveal the fate of hematopoietic stem cells influenced by Flk2$^-$ multipotent progenitors after transplantation

Zheng Wang[1,2], Du Jiang[1], Mary Vergel[1], Anna Nogalska[1], Rong Lu[1*]

[1] Department of Stem Cell Biology and Regenerative Medicine, Eli and Edythe Broad Center for Regenerative Medicine and Stem Cell Research, Keck School of Medicine, University of Southern California, Los Angeles, CA 90033;

[2] Current address: Medical Center of Hematology, State Key Laboratory of Trauma, Burns and Combined Injury, Xinqiao Hospital, Third Military Medical University, Chongqing, China.

[*] To whom correspondence should be addressed. Email: **ronglu@usc.edu**

Supplementary Figure 1

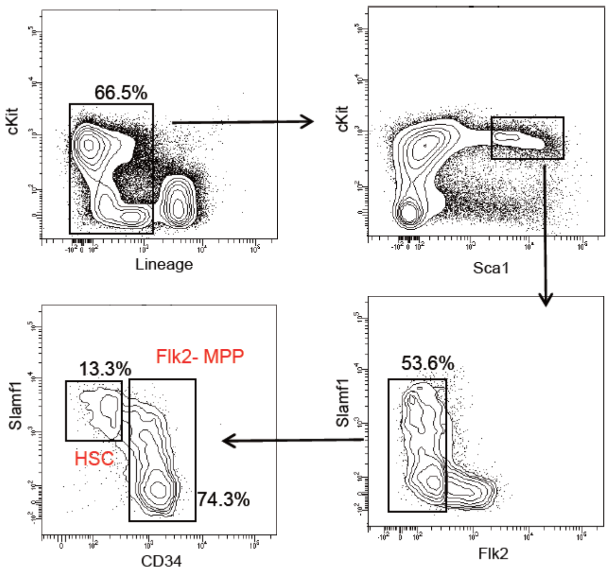

Supplementary Fig. 1. FACS gating strategy for donor hematopoietic stem cells and MPPs isolation.

FACS gating for sorting HSCs (lineage (CD3, CD4, CD8, B220, Gr1, Mac1, Ter119)$^{-}$ /cKit^{+} /Sca1^{+} /Flk2^{-} /CD34^{-}/CD150^{+}) and MPPs (lineage (CD3, CD4, CD8, B220, Gr1, Mac1, Ter119)$^{-}$ /IL7Ra^{-}/cKit^{+}/Sca1^{+}/Flk2^{-}/CD34^{+}) from cKit enriched bone marrow cells.

Supplementary Figure 2

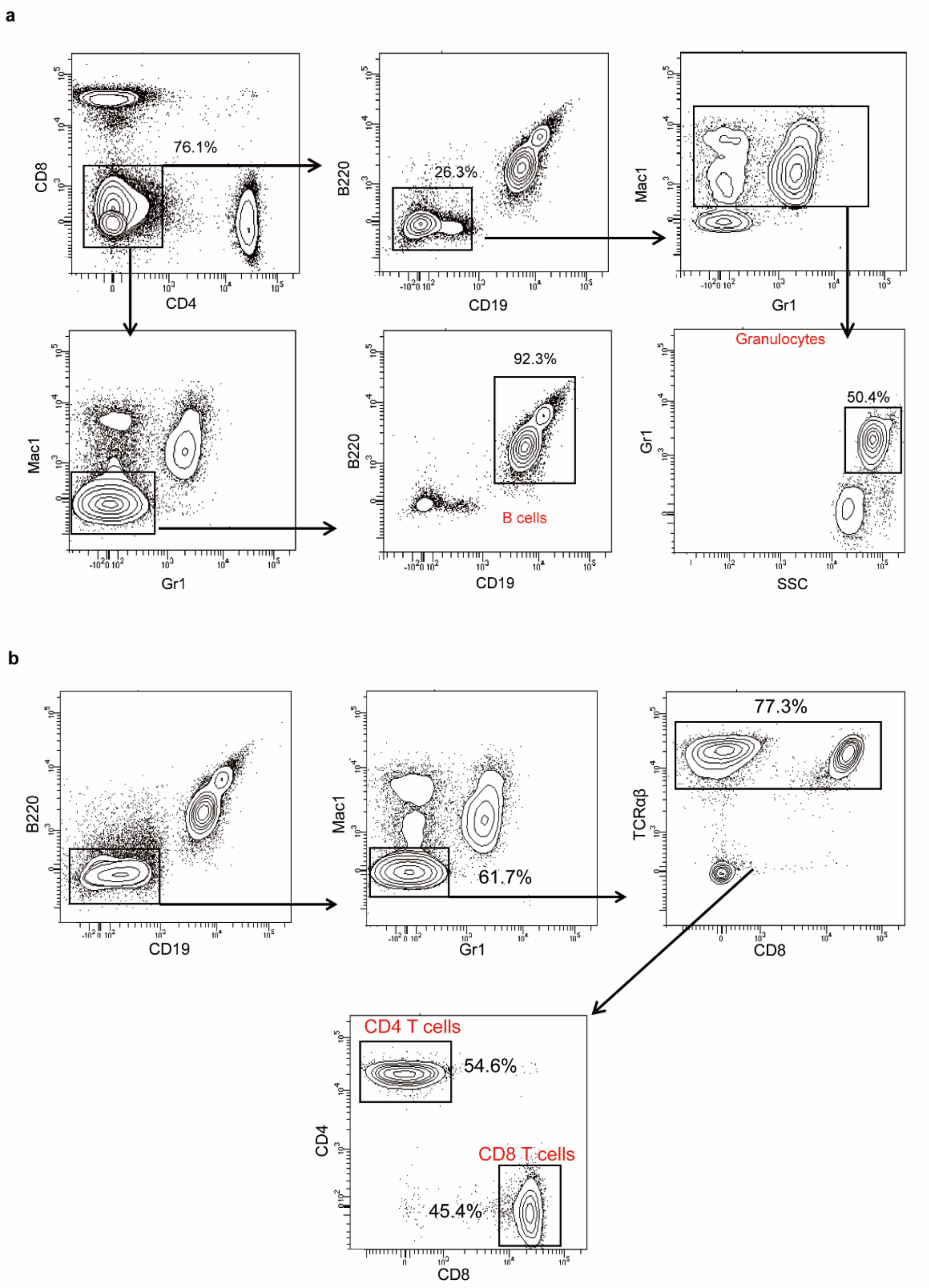

Supplementary Fig. 2. FACS gating strategy for sorting peripheral blood cells.

FACS gating for sorting four blood cell populations highlighted in red, **(a)** granulocytes and B cells, **(b)** CD4T and CD8T cells. Red blood cells were lysed before sorting (details described in Methods). A summary of all the cell surface markers for each harvested cell population is included in Methods.

Supplementary Figure 3

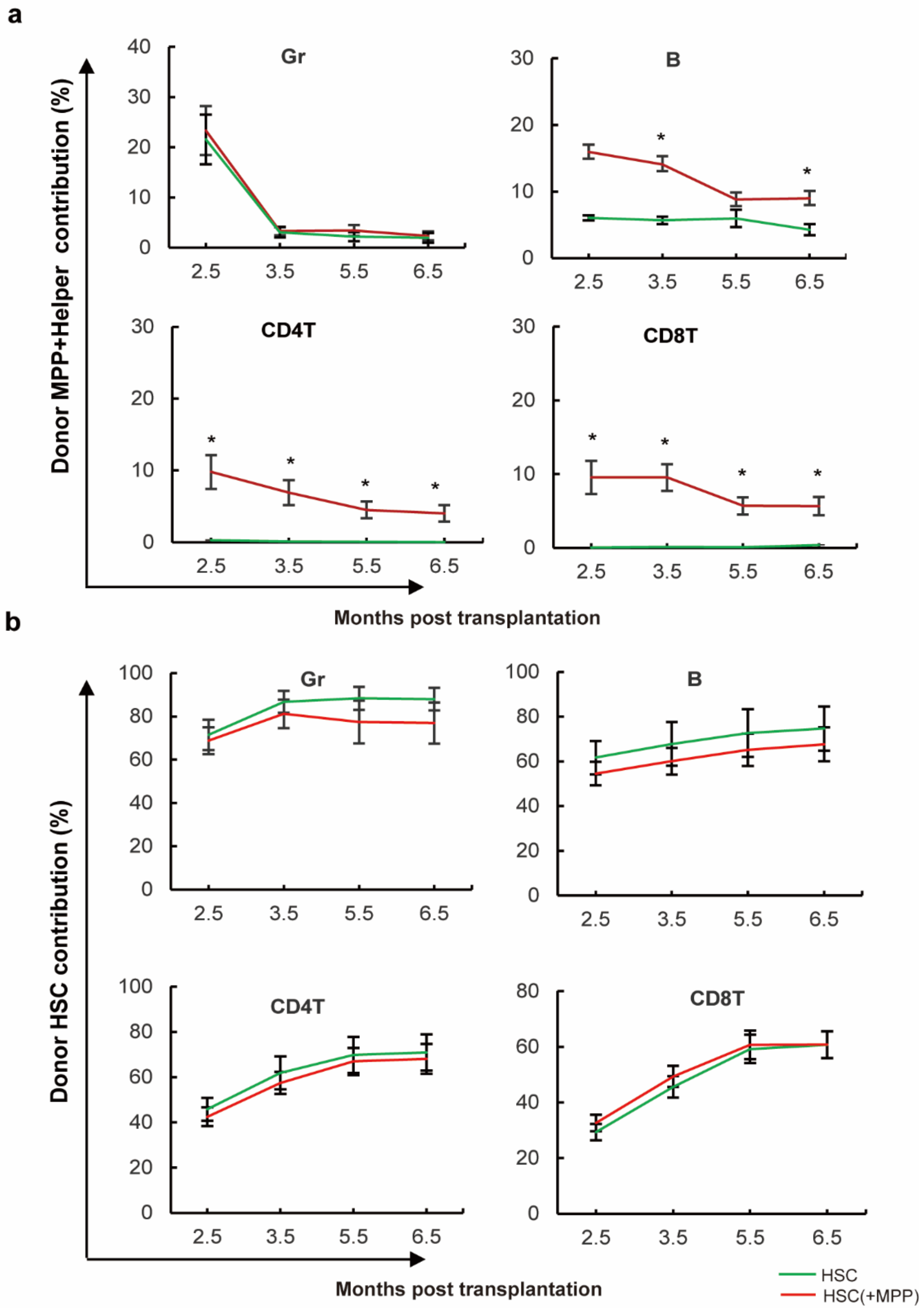

Supplementary Fig. 3. Donor chimerism post-transplantation.

Flow cytometry analysis of **(a)** helper and donor MPPs chimerism, and **(b)** donor HSC chimerism in granulocytes, B, CD4T, and CD8T cells at different time points post-transplantation. Data are shown as mean ± SEM. n=5 mice for the HSC only group (HSC) and n=7 mice for the HSC co-transplanted with MPP group (HSC(+MPP)) for the population level analysis. *P ≤ 0.05, one-tailed Student’s t-test.

Supplementary Figure 4

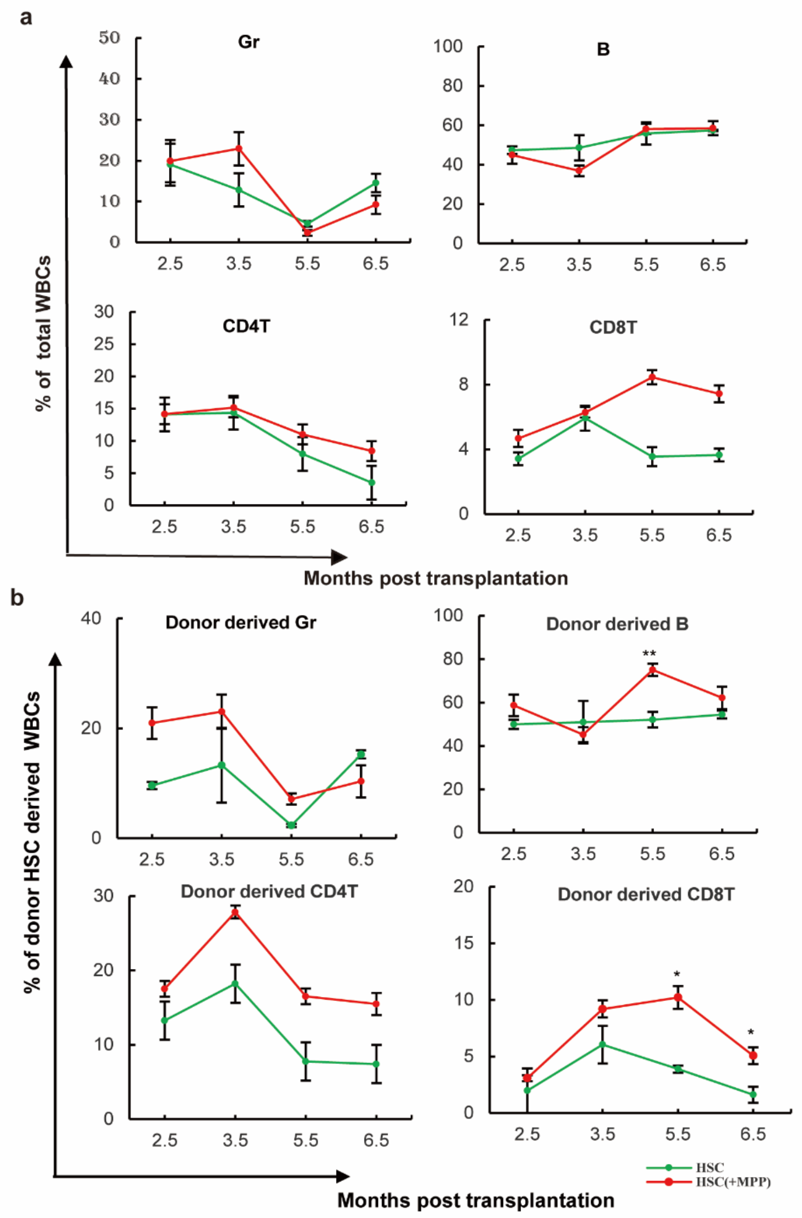

Supplementary Fig. 4. The dynamics of blood production after MPP co-transplantation in a replicate experiment.

(a) Abundance of granulocytes (Gr), B, CD4T and CD8T cells in the peripheral blood of the recipient mice. **(b)** Abundance of granulocytes (Gr), B, CD4T and CD8T cells among donor-derived white blood cells (WBCs). Data are shown as mean ± SEM, *P ≤ 0.05, **P < 0.01 by one-tailed Student's t-test. For this replicate experiment, n=5 mice for the HSC only group (HSC), and n=9 mice for HSC co-transplanted with MPP group (HSC(+MPP)).

Supplementary Figure 5

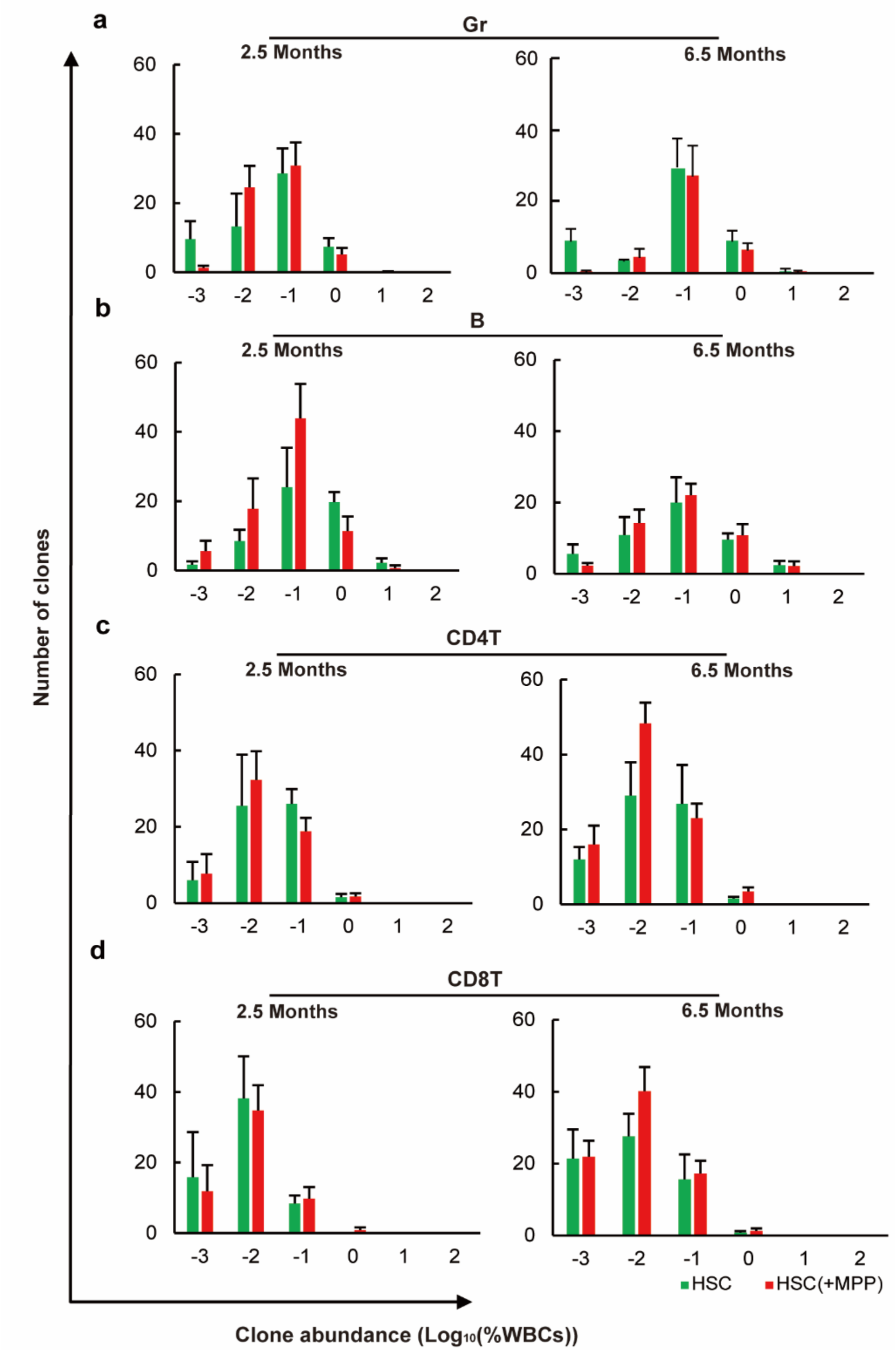

Supplementary Fig. 5. The clonal abundance distribution for each type of blood cell.

Number of HSC clones that produce different amounts of blood cells including Gr (**a**), B (**b**), CD4T (**c**) and CD8T (**d**). Data are shown as mean ± SEM. n=4 mice for the HSC only group (HSC) and n=6 mice for the HSC co-transplanted with MPP group (HSC(+MPP)) for the clonal level analyses of all figures.

Supplementary Figure 6

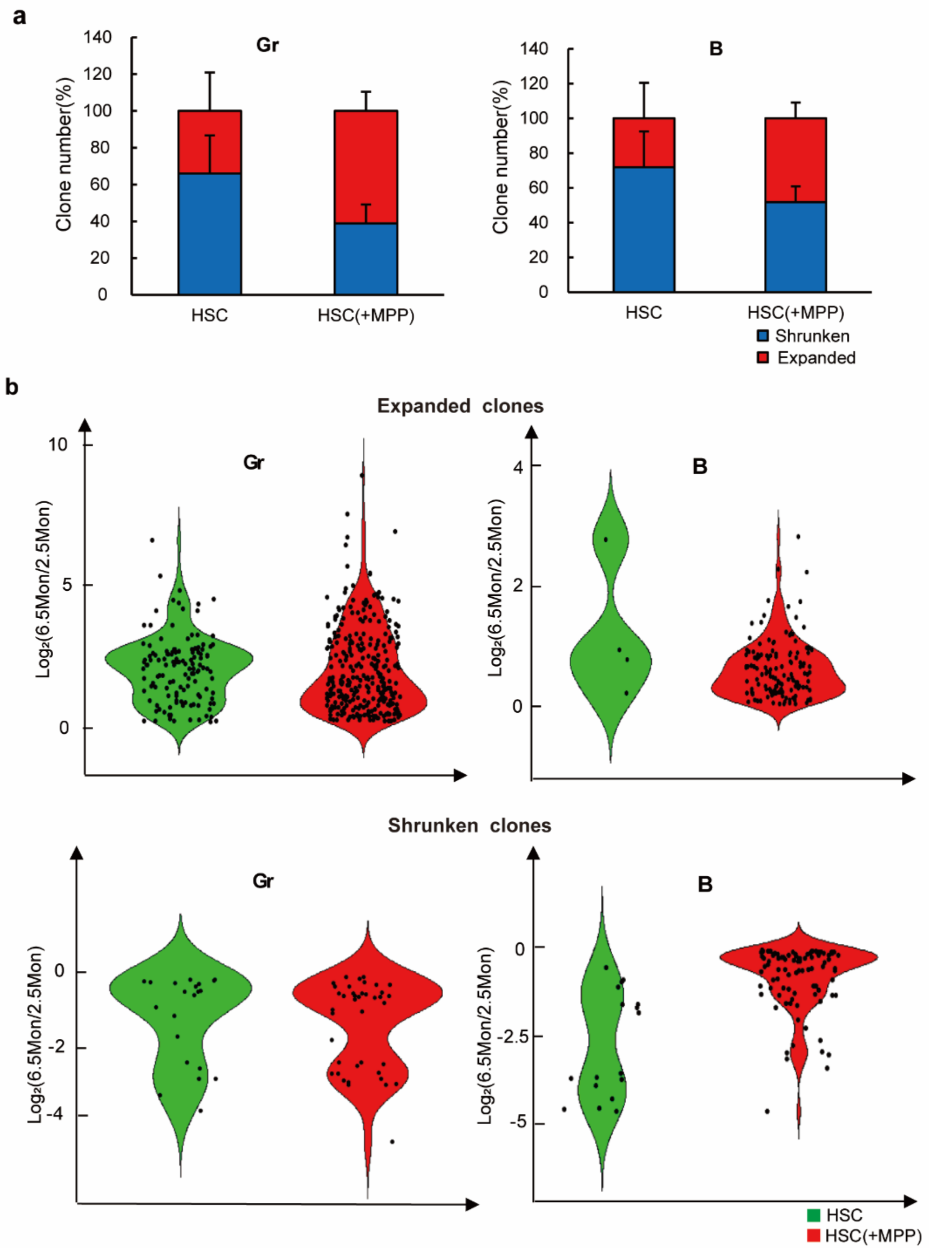

Supplementary Fig. 6. Changes of the HSCs clones producing granulocytes (Gr) or B cells over time.

(a) Proportion of “shrunken” and “expanded” clones among all persisted clones that produce Gr or B cells. The ratio of CD4T or CD8T cell production at 6.5 months versus 2.5 months post-transplantation defined clones as “shrunken” (below 1) and “expanded” (above 1). Data are shown as mean ± SEM. **(b)** Clonal abundance changes over time for “expanded” or “shrunken” clones that produce Gr or B cells. Each dot represents one clone.

Supplementary Figure 7

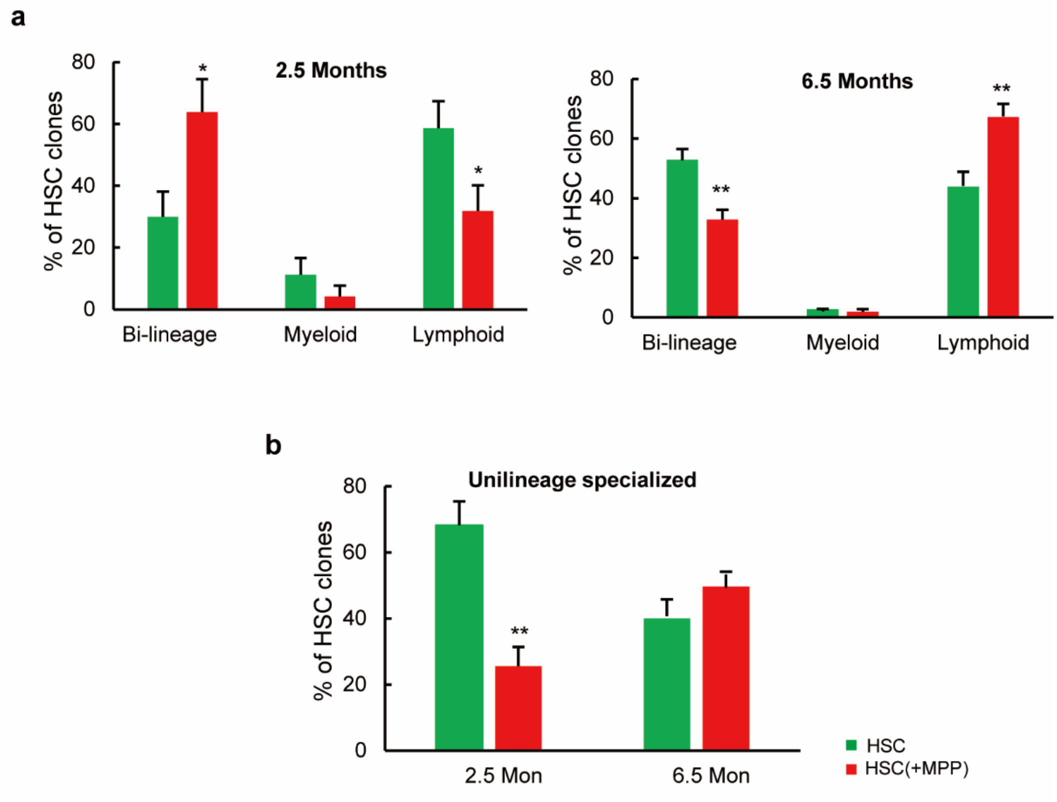

Supplementary Fig. 7. MPP co-transplantation changes lineage preferences of HSC differentiation.

(a) Number of HSC clones producing blood cells of myeloid lineage (granulocytes) and lymphoid lineage (B and T cells), or both lineages (Bi-lineage). **(b)** Number of HSC clones producing blood cells of either myeloid lineage (granulocytes) or lymphoid lineage (B and T cells). * $P \leq 0.05$, ** $P < 0.01$, one-tailed Student's t-test. Data are shown as mean ± SEM.

Supplementary Figure 8

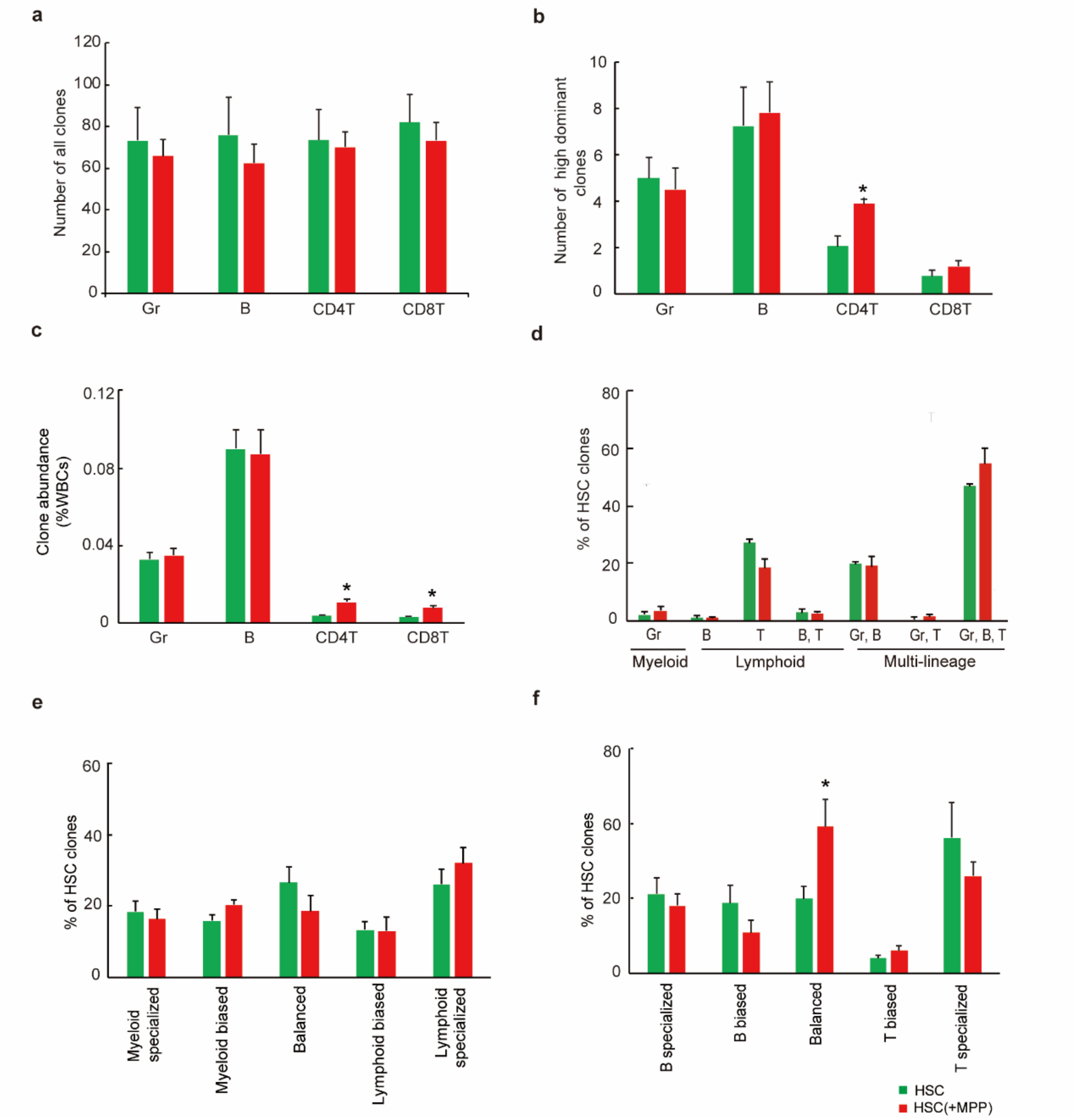

Supplementary Fig. 8. Characterizing blood production of HSC clones at 5.5 months post-transplantation

(a) Number of clones producing granulocyte (Gr), B, CD4T, and CD8T cells at 5.5 months post-transplantation, related to **Fig. 3a**. **(b)** Number of high abundance clones (above 0.05% of WBCs) producing each cell type, related to **Fig. 3b**. **(c)** Average abundance of clones producing each cell type, related to **Fig. 3c**. **(d)** Number of HSC clones producing different cell types. Clones are categorized by the combination of cell types that they produce at 2.5 or 6.5 months post-transplantation. All combinations of measured cell types are displayed in the graph. Clones that produced CD4T or CD8T cells were combined as T cell producing clones. Related to **Fig. 4a.** (**e**) Number of HSC clones exhibiting various lineage biases. Lineage-biased clones are defined as

those whose relative copy numbers in one lineage are 2.4142 (cotangent 22.5 degrees) times more than their relative copy numbers in the other lineage. Lineage-specialized clones are defined as clones producing measured lymphoid or myeloid cells, but not cells of both lineages. Related to **Fig. 4b**. (**f**) Number of HSC clones exhibiting various biases in producing B cells and T cells, related to **Fig. 4c**. Data shown as mean ± SEM. *: $P \leq 0.05$, one-tailed Student's t-test.

Supplementary Table 1

The total number of HSC clones contributing to each blood cell type at different time points post-transplantation. Each row shows data from one mouse.

Mouse	2.5 Months				5.5 Months				6.5 Months			
	Gr	B	CD4T	CD8T	Gr	B	CD4T	CD8T	Gr	B	CD4T	CD8T
HSC_01	33	39	128	163	97	106	132	139	67	80	148	142
HSC_02	122	88	41	59	76	71	68	75	56	57	80	75
HSC_03	56	37	45	46	50	56	41	53	32	14	56	54
HSC_04	52	62	25	34	61	61	45	55	75	55	39	46
HSC(+MPP)_01	73	73	72	71	48	56	60	67	36	59	75	74
HSC(+MPP)_02	113	126	108	108	88	78	103	103	39	50	124	122
HSC(+MPP)_03	79	138	59	75	68	72	80	85	51	75	141	129
HSC(+MPP)_04	57	83	56	62	70	67	70	73	76	41	98	91
HSC(+MPP)_05	54	74	53	56	54	62	68	63	35	37	91	85
HSC(+MPP)_06	36	42	27	30	48	32	62	60	35	58	73	55

Supplementary Table 2

Shannon diversity index for each cell type at different time points post-transplantation. Each row shows data from one mouse.

Mouse	2.5 Months				5.5 Months				6.5 Months			
	Gr	B	CD4T	CD8T	Gr	B	CD4T	CD8T	Gr	B	CD4T	CD8T
HSC_01	4.273	4.296	4.246	4.219	4.016	4.338	4.290	4.291	3.499	4.090	4.303	4.267
HSC_02	4.717	4.831	4.607	4.586	4.503	4.524	4.792	4.801	3.610	3.984	4.803	4.779
HSC_03	4.353	4.918	4.068	4.280	4.358	4.480	4.988	4.883	3.842	4.365	4.922	4.831
HSC_04	4.041	4.401	4.004	4.093	4.362	4.239	4.561	4.587	3.910	3.767	4.564	4.466
HSC(+MPP)_01	3.967	4.286	3.965	4.016	4.115	4.206	4.536	4.512	3.375	3.648	4.472	4.395
HSC(+MPP)_02	3.555	3.703	3.205	3.286	4.044	4.162	4.298	4.251	3.235	4.017	4.258	3.966
HSC(+MPP)_03	3.407	3.658	4.795	5.041	4.703	4.896	5.080	5.038	4.564	4.450	4.937	4.853
HSC(+MPP)_04	4.776	4.532	3.676	3.955	4.186	4.320	4.299	4.512	4.098	4.284	4.334	4.280
HSC(+MPP)_05	4.012	3.590	3.788	3.819	3.348	3.454	3.972	4.080	3.398	3.124	4.012	3.923
HSC(+MPP)_06	3.805	4.173	3.038	3.417	3.933	4.104	3.542	3.997	4.162	4.199	3.615	3.790
